# Protein disulfide isomerase as a prosurvival factor in cell therapy for muscular and vascular diseases

**DOI:** 10.1186/s13287-018-0986-y

**Published:** 2018-09-26

**Authors:** Giuliana Di Rocco, Silvia Baldari, Antonietta Gentile, Maurizio Capogrossi, Gabriele Toietta

**Affiliations:** 1 Department of Research, Advanced Diagnostic and Technological Innovation, IRCCS Regina Elena National Cancer Institute, via E. Chianesi 53, 00144 Rome, Italy; 2 Vascular Pathology, IRCCS Istituto Dermopatico dell’Immacolata, via dei Monti di Creta 104, 00167 Rome, Italy; 3 Present address: Department of Systems Medicine, Synaptic Immunopathology Laboratory, University of Rome Tor Vergata, Via Montpellier 1, 00133 Rome, Italy

**Keywords:** Cell and tissue-based therapy, Cell survival, Duchenne muscular dystrophy, Endoplasmic reticulum stress, Endothelial cells, Ischemia, Molecular chaperones, Myoblasts, Protein disulfide isomerase, Regenerative medicine

## Abstract

**Background:**

Cell therapy for degenerative diseases aims at rescuing tissue damage by delivery of precursor cells. Thus far, this strategy has been mostly unsuccessful due to massive loss of donor cells shortly after transplantation. Several strategies have been applied to increase transplanted cell survival but only with limited success. The endoplasmic reticulum (ER) is an organelle involved in protein folding, calcium homeostasis, and lipid biosynthesis. Protein disulfide isomerase (PDI) is a molecular chaperone induced and activated by ER stress. PDI is induced by hypoxia in neuronal, cardiac, and endothelial cells, supporting increased cell survival to hypoxic stress and protection from apoptosis in response to ischemia.

**Methods:**

We achieved ex vivo PDI gene transfer into luciferase-expressing myoblasts and endothelial cells. We assessed cell engraftment upon intramuscular transplantation into a mouse model of Duchenne muscular dystrophy (*mdx* mouse) and into a mouse model of ischemic disease.

**Results:**

We observed that loss of full-length dystrophin expression in *mdx* mice muscle leads to an increase of PDI expression, possibly in response to augmented ER protein folding load. Moreover, we determined that overexpression of PDI confers a survival advantage for muscle cells in vitro and in vivo to human myoblasts injected into murine dystrophic muscle and to endothelial cells administered upon hindlimb ischemia damage, improving the therapeutic outcome of the cell therapy treatment.

**Conclusions:**

Collectively, these results suggest that overexpression of PDI may protect transplanted cells from hypoxia and other possibly occurring ER stresses, and consequently enhance their regenerative properties.

## Background

Maintenance and regeneration of skeletal muscles mainly depend on resident stem cells known as satellite cells. The satellite cell pool that takes part in myofiber repair is progressively exhausted with age [1] and in muscle degenerative disorders characterized by repetitive cycles of muscle degeneration and regeneration, such as Duchenne muscular dystrophy (DMD) [2]. Cell therapy approaches for degenerative muscle diseases aim at rescuing muscle damage by delivery of cells able to differentiate into skeletal muscle [3, 4]. Satellite cells represent the primary choice for cell-based therapy due to their commitment to the myogenic lineage [5, 6]. However, satellite cells from muscle biopsies are recovered in low numbers, grow poorly in vitro, and rapidly undergo senescence [7] as a consequence of replicative aging associated with telomere shortening [8]. The reduced capacity to proliferate during the step of in vitro amplification hampers the clinical translation of possible satellite cell-based therapies for DMD [3, 9]. In addition, cellular polarity is lost in dystrophin-deficient satellite cells, leading to asymmetric cell division, improper differentiation, and consequently to an overall reduction in the number of myogenic progenitor cells [6]. To date, myoblast transplantation treatment has been generally ineffective given that the functional support of engrafted cells is limited due to massive donor cell death shortly after transplantation [10–12]. Several studies have shown that freshly isolated, uncultured satellite cells, as well as satellite cells still enclosed in their myofiber niche, regenerate muscle much more efficiently than cells exposed to culture conditions, so that very low numbers of such cell are necessary for regenerative purposes compared to cultured cells [13, 14]. However, genetic correction of mutated cells, as would be required in the case of a homologous transplant, requires ex vivo culture and expansion. Therefore, further studies aimed at defining more valid strategies to prevent myoblast death in the early stage after transplantation are required [15]. Direct muscle injection exposes transplanted cells to prolonged periods of hypoxia, particularly exacerbated in dystrophic muscles which are characterized by high levels of reactive oxygen species (ROS) [16].

Loss of function mutations in the gene encoding for the 427-kDa cytoskeletal protein dystrophin cause DMD. Dystrophin has a structural role in muscle, connecting the cytoskeleton to the basal lamina. When functional dystrophin is absent, skeletal muscle signaling is disrupted, leading to progressive damage and membrane leakage, to fiber degeneration and necrosis [17]. The *mdx* mouse harbors a point mutation in the dystrophin gene and is considered a surrogate model for DMD [18]. Interestingly, the full-length and shorter isoforms of dystrophin are highly transcribed in the satellite cells from wild-type and *mdx* mice, respectively [19]. Unfolded fragments of dystrophin produced from the *mdx* premature termination codon accumulate in the endoplasmic reticulum (ER)/Golgi compartments triggering ER stress, resulting in activation of the unfolded protein response (UPR) [20]. To counteract the accumulation of unfolded proteins, UPR activation leads to upregulation of ER resident chaperones, reduction of protein translation, and increase in the degradation of unfolded proteins [21]. However, if the stress is severe and/or prolonged, the ER also initiates apoptotic signaling and promotes production of ROS [22]. Thus, ER stress response has relevant implications in deciding cell survival or death [23]. Remarkably, the rate of accumulation of unfolded proteins is likely to be much higher in satellite cells than in cells with a higher turnover rate, making satellite cells more exposed to proteotoxicity linked to altered protein homeostasis [24].

Protein disulfide isomerase (PDI) and its related family members are among the ER chaperones upregulated upon UPR activation [25]. PDI has two enzymatic activities: as an oxidoreductase, it can catalyze the formation, reduction, and isomerization of disulfide bonds; and as a polypeptide binding protein, it works as a molecular chaperone supporting the folding of nascent polypeptides, consequently increasing the yield of correctly folded protein molecules [26, 27]. Disulfide bond formation and proper protein folding occur in the ER. In addition, PDI has a copper binding activity which plays a key role in regulating intracellular disposition of this redox-active metal; PDI may also control the function of certain extracellular matrix proteins by regulating their redox state [28]. PDI prevents neurotoxicity associated with ER stress and protein misfolding in neurodegenerative disorders such as Parkinson’s or Alzheimer’s disease [29]. Upregulation of PDI in response to hypoxia has been demonstrated in neuronal, cardiac, and endothelial cells. Overexpression of PDI in these cells results in an increase of cell viability in response to hypoxia and protection from apoptosis in response to ischemia [30]. However, the possible involvement of ER stress-associated proteins, and in particular of molecular chaperones such as PDI, in the skeletal muscle system and in its degenerative pathologies has been only partially investigated [31].

In this report we evaluated PDI expression in skeletal muscle of *mdx* mice in comparison with their wild-type counterpart. Moreover, we tested the hypothesis that viral-mediated overexpression of PDI might be instrumental in promoting survival and engraftment of primary myoblasts transplanted into *mdx* mice, possibly increasing the therapeutic efficacy of the procedure. Furthermore, we evaluated a similar strategy to promote a cell therapy intervention aimed at promoting angiogenesis in a mouse model of hindlimb ischemia.

## Methods

### Experimental animal procedures

Procedures involving living animals were approved by local ethics committees and were performed according to the Guidelines of the Italian National Institutes of Health (Art. 31 D.lgs 26/2014, 4 March 2014). Animals used in the study were 3-month-old dystrophic C57BL/ 10ScSn Dmd^*mdx*^ and age-matched wild-type control mice provided by Charles River (Calco, Lecco, Italy). Postoperatively, animals were administered by intraperitoneal injection of the clinically approved immunosuppressive drug tacrolimus (FK-506; Sigma-Aldrich St. Louis, MO, USA) 2 mg/kg per day [32]. Acute hindlimb ischemia was induced by removal of the femoral artery, as described previously [33]. Measure of the blood flow in the ischemic hindlimb compared to the contralateral control was performed by laser Doppler perfusion imaging (Lisca Inc., North Brunswick, NJ, USA).

### Cell culture

Human primary myoblasts were obtained from ThermoFisher Scientific (Waltham, MA, USA) and cultured according to the manufacturer’s instructions. Human endothelial cells were isolated from adipose tissue collected during cosmetic surgery procedures, as described previously [34]. Each subject gave her/his written informed consent to use harvested adipose tissue samples for research purposes. The study protocol was approved by the Institutional Review Board (n° 1794/15, 13/02/2015) and was performed in accordance with the principles of Good Clinical Practice expressed in the Declaration of Helsinki. The C2C12 immortalized muscle cell line derived from CH3 wild-type mice were cultured in accordance with the American Type Culture Collection specifications in Dulbecco’s modified Eagle’s medium (DMEM; Gibco, Grand Island, NY, USA) supplemented with 10% (v/v) fetal bovine serum (FBS) and 1% (v/v) penicillin–streptomycin solution (50 U/ml penicillin and 50 μg/ml streptomycin), at 37 °C in a humidified atmosphere of 95% air and 5% CO_2_. Before administration cells were counted, resuspended in 25 μl of phosphate-buffered saline (PBS), and delivered into the tibialis anterior muscle of the experimental animals.

### Endoplasmic reticulum stress induction and cell proliferation analysis

Tunicamycin, a mixture of antiviral nucleoside antibiotics; thapsigargin, an inhibitor of the ubiquitous sarcoplasmic reticulum/endoplasmic reticulum Ca^++^ ATPase; and MG132, a specific proteasome inhibitor that blocks ER-associated protein degradation, are commonly used as pharmaceutical ER stress inducers [35]. C2C12 cells (2 × 10^5^ cells) were plated on 60-mm dishes and cultured in medium supplemented with either 5.0 μg/μl tunicamycin, 0.5 μM thapsigargin, or 10.0 μM MG132 (all from Sigma-Aldrich) for 6 h at 37 °C. Cell proliferation after treatment was measured using the WST-1 cell proliferation assay kit (Takara, Clontech, Mountain View, CA, USA), according to the manufacturer’s instructions. The optical density at 450 nm was assessed using a microplate reader (BioRad Laboratories Inc., Hercules, CA, USA). All experiments were performed at least twice in duplicate, and the relative cell viability (%) was expressed as a percentage relative to the untreated control cells.

The plant flavonoid quercetin has protective effects on ER stress in intestinal epithelial cells [36]. On this basis, C2C12 cells were treated with quercetin (75 μM) (Sigma-Aldrich) for 24 h and then challenged either with tunicamycin (0.3 μM) or thapsigargin (1.5 μg/ml) for an additional 16 h before collection for western blot analysis and cell proliferation assay.

### Viral vector production and viral-mediated gene transfer

Recombinant E1–E3-deleted adenoviral vectors expressing GFP used as control or PDI and GFP (PDI-GFP) were produced as described previously [30]. Third-generation, self-inactivating, recombinant vesicular stomatitis virus-pseudotyped lentiviral vectors (LV) expressing firefly luciferase were obtained as reported previously [37]. Viral-mediated gene transfer in human primary myoblasts and endothelial cells was performed as described previously [32, 37]. Transduction of C2C12 cells was achieved with transfection with Lipofectamine LTX (ThermoFisher Scientific).

### Immunoblotting analysis

For western blot analysis, cells (1.5 × 10^5^ cells/60-mm dish) were collected and protein lysates were loaded on polyacrylamide gel. Following SDS-PAGE, immunoblot analysis was performed according to established protocols using the following primary antibodies: anti-poly(ADP-ribose) polymerase (PARP), anti-binding immunoglobulin protein (GRP78/BiP), endoplasmic reticulum oxidoreductin-1α (ERO1α), anti-PDI (Cell Signaling Technology, Danvers, MA, USA), and anti-p21 (Santa Cruz Biotechnology, Dallas, TX, USA). Glyceraldehyde phosphate dehydrogenase (GAPDH) has been used as the protein loading normalization. Densitometry analysis was performed using ImageJ software (National Institutes of Health, Bethesda, MD, USA).

### Optical bioluminescent imaging

For in vivo bioluminescent imaging (BLI) analysis, mice were anesthetized and d-luciferin dissolved in PBS (150 mg/kg body weight) was administered by intraperitoneal injection. Analysis was performed using the IVIS Lumina II instrument equipped with Living Image software for data quantification (PerkinElmer, Waltham, MA, USA), according to an established procedure [38].

### Immunohistochemistry

At necropsy, hindlimb muscles were dissected, fixed in formalin for 48 h, and embedded in paraffin, as described previously [39]. Immunohistochemistry on deparaffinized sections was performed using the following antibodies: rabbit polyclonal antibody anti-GFP (Ab290, 10 μg/ml; Abcam, Cambridge, UK), rabbit polyclonal anti-carboxy-terminal portion of dystrophin (Ab15277, 2 μg/ml; Abcam), rabbit polyclonal antibody anti-PDI H-160 (1:200 dilution; Santa Cruz Biotechnology), and rabbit polyclonal antibody against firefly luciferase (1:500 dilution; Sigma Aldrich). For immunofluorescence studies, sections were processed with fluorochrome-conjugated anti-rabbit antibody (1:40 dilution; Dako/Agilent Technologies, Santa Clara, CA, USA), stained with Hoechst to identify nuclei, and mounted in Vectashield (Vector Laboratories, Burlingame, CA, USA). Images were acquired with a fluorescence microscope equipped with image analyzer KS300 software (Carl Zeiss, Oberkochen, Germany).

### Statistical analysis

Results are expressed as mean ± standard error of the mean. Data analysis and comparisons between groups were done with INSTAT software (GraphPad, San Diego, CA, USA). The significance of differences was assessed with a two-tailed Student *t* test for unpaired data; statistical significance was set at *p* < 0.05.

## Results

### Protein disulfide isomerase levels are increased in *mdx* versus wild-type skeletal muscle

**Fig. 1:**
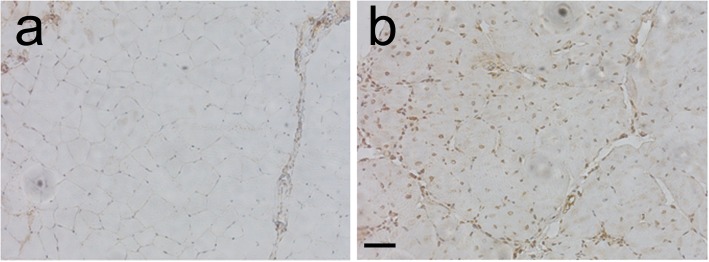
PDI expression in tibialis anterior muscle in wild-type and *mdx* mice. Immunohistochemical staining using anti-PDI specific antibodies on representative sections of tibialis anterior muscles isolated from wild-type (**a**) and *mdx* (**b**) mice. Scale bar 50 μm

The *mdx* mouse is the most common animal model used in DMD research. In the *mdx* mouse, the dystrophin gene contains a premature stop codon in exon 23; the lack of full-length dystrophin protein is linked with higher protein turnover [40], with a compensatory higher expression of utrophin [41], filamin 2, cytoplasmic γ-actin [42], and α7β1 integrin [43]. Accumulation of truncated, misfolded dystrophin in the ER and enhanced expression of several other proteins, some of which, such as the β1 integrin subunit, are naturally rich in disulfide bonds, may result in upregulation of ER chaperones like PDI. In skeletal muscle, resident ER chaperones, in addition to occupying ER perinuclear regions, are distributed within all of the sarcoplasmic reticulum [44]. To assess the level of expression and the localization of PDI in muscle sections of wild-type and *mdx* mice, we performed immunohistochemistry using a specific anti-PDI antibody. We revealed that PDI expression is stronger in *mdx* regenerating muscle (Fig. 1), suggesting a possible induction of ER stress caused by accumulation of the misfolded truncated form of dystrophin [20]. This result is consistent with previous reports indicating increased levels of some ER stress markers, including GRP78, PERK, eIF2a, IRE1, sXBP1 [45], and CHOP [46] in skeletal muscle from dystrophic versus wild-type mice. Therefore, upregulation of PDI expression in *mdx* muscle may represent a cellular response to the pathological condition aiming at preserving protein homeostasis [45].

### Endoplasmic reticulum stress induction in vitro reduces muscular cell viability

**Fig. 2:**
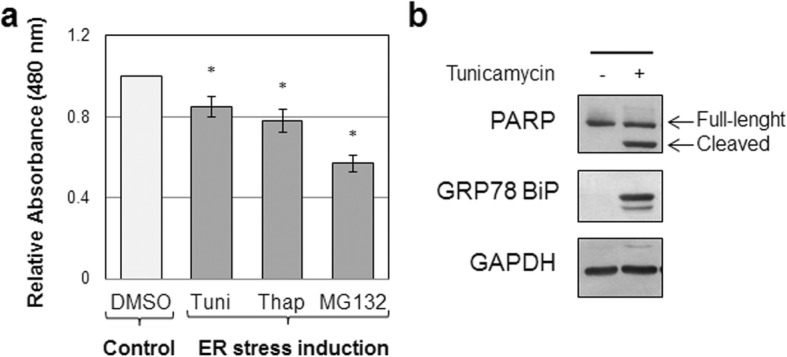
ER stress induction affects C2C12 cell proliferation and survival. C2C12 immortalized mouse myoblast cell line (1.5 × 10^3^ cells/60-mm dish) treated with either tunicamycin (Tuni) (5 μg/ml), thapsigargin (Thap) (0.3 μM), MG132 (10 μM), or vehicle solution only (DMSO) as control for 6 h. **a** Cell proliferation measured using WST-1 cell proliferation assay kit. Results reported as mean ± standard deviation of three independent experiments performed in duplicate. *p < 0.005. **b** C2C12 cells treated with tunicamycin for 16 h and immunoblot analysis performed using specific antibodies against GRP78/BiP and PARP (full-length and cleaved form) to assess ER stress and apoptotic cell death induction, respectively. GAPDH used as protein loading normalization. DMSO dimethylsulfoxide, ER endoplasmic reticulum, GAPDH glyceraldehyde phosphate dehydrogenase, GRP78/BiP anti-binding immunoglobulin protein, PARP anti-poly(ADP-ribose) polymerase

We investigated the effects of ER stress exposure into the murine mouse myoblast C2C12 cell line, a widely used model to investigate in vitro myoblast proliferation and differentiation. We performed a cell proliferation assay on C2C12 cells upon ER stress induced by tunicamycin, thapsigargin, and MG132. We observed that the treatments affected short-term proliferation and survival of C2C12 cells (Fig. 2a), thus indicating that ER stress determined a reduction of muscular cell proliferation and viability. In addition, in response to ER stress induced by tunicamycin treatment we observed by immunoblot analysis a marked activation of the binding immunoglobulin protein (GRP78/BiP), a marker of ER stress, and the cleavage of poly(ADP-ribose) polymerase (PARP), a hallmark of apoptosis (Fig. 2b). Thus, under ER stress conditions, muscular cells activate GRP78/BiP overexpression aiming at rescuing cells from the effects of accumulation of misfolded proteins. Acute and unresolved ER stress may lead to apoptotic cell death. Alleviation of ER stress by may therefore help in reestablishing ER homoeostasis, reducing apoptosis.

### Protein disulfide isomerase overexpression has prosurvival effects upon endoplasmic reticulum stress induction in vitro

**Fig. 3:**
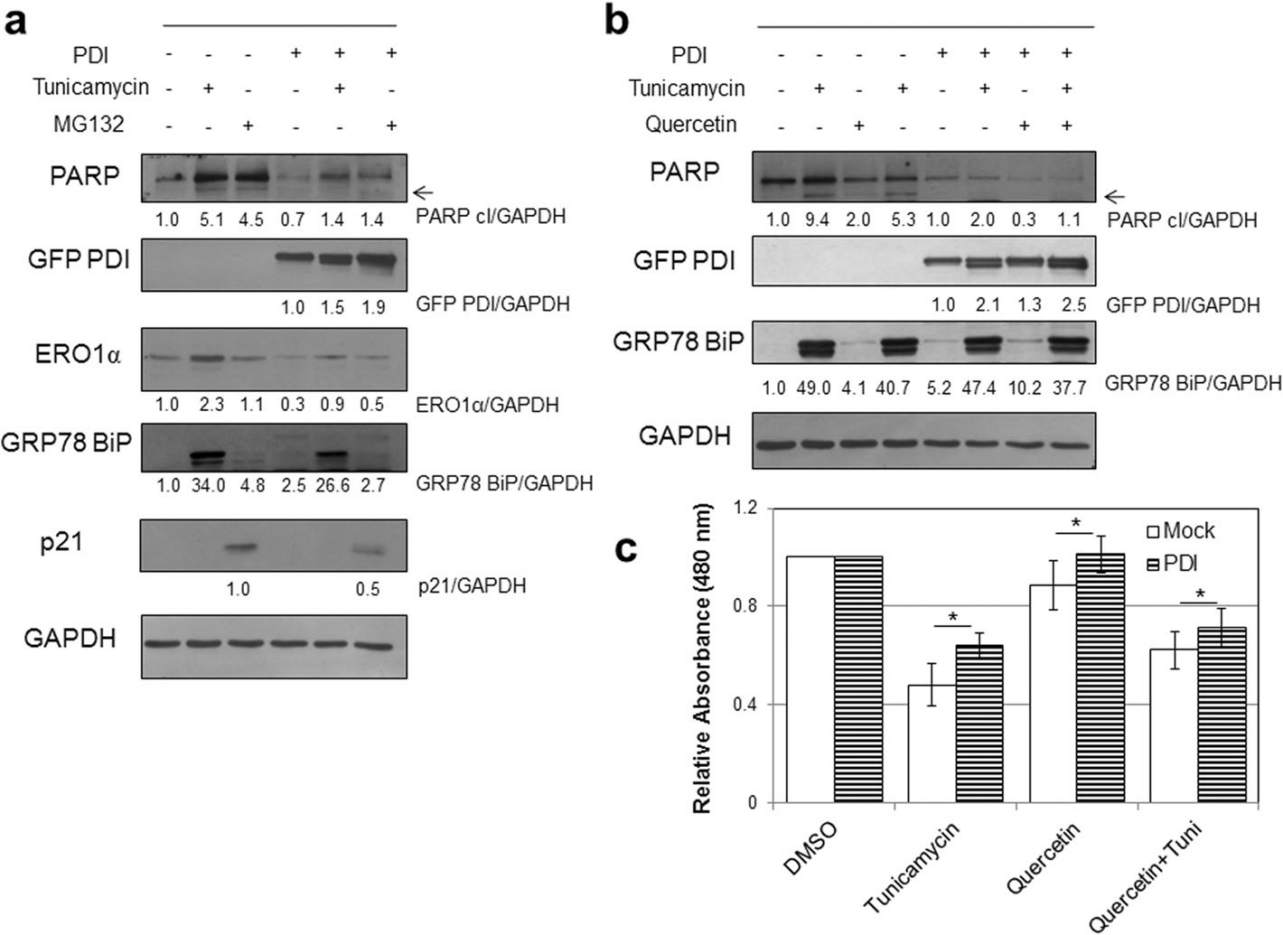
Protective effect of PDI overexpression and quercetin treatment on ER stress activation, apoptotic cell death, and cell survival. **a** C2C12 cells (1 × 10^3^ cells/96-well plates) underwent PDI gene transfer 24 h before treatment either with tunicamycin (5 μg/ml) or MG132 (10 μM) for 6 h. Protein lysates (30 μg/lane) analyzed by immunoblot analysis using specific antibodies against PARP (full-length and cleaved form) to assess apoptotic cell death induction. GRP78/BiP and ERO1α assessed as ER stress markers and p21 as cellular senescence marker; antibodies against Turbo GFP used to verify GFP/PDI overexpression. GAPDH used as protein loading normalization. Densitometry performed using ImageJ software and relative band intensities of tunicamycin, and MG132-treated cells normalized to GAPDH and finally quantified with respect to untreated control, arbitrarily set to 1.0. **b** Mock transfected and PDI-overexpressing C2C12 cells preconditioned with quercetin (75 μM) for 24 h and then challenged with ER stress-inducer tunicamycin (Tuni). Immunoblot and densitometric analysis performed as already described, while (**c**) cell viability evaluated using WST-1 cell proliferation assay kit. Results reported as mean ± standard deviation of three independent experiments performed in duplicate. *p < 0.005. DMSO dimethylsulfoxide, ERO1α endoplasmic reticulum oxidoreductin-1α, GAPDH glyceraldehyde phosphate dehydrogenase, GFP green fluorescent protein, GRP78/BiP anti-binding immunoglobulin protein, PARP anti-poly(ADP-ribose) polymerase, PDI protein disulfide isomerase

To analyze the potential protective role of PDI in cells under ER stress, we evaluated the effect of PDI overexpression in C2C12 cells. To this aim, cells were transfected with a PDI-GFP-expressing vector and transduction was confirmed using anti-turboGFP specific antibody immunoblot (Fig. 3a). As previously determined (Fig. 2b), ER stress induction in C2C12 muscle cells caused a marked activation of the markers of ER stress GRP78/BiP and endoplasmic reticulum oxidoreductin-1α (ERO1α) and increased levels of the apoptotic marker PARP. Consistently, PDI-overexpressing cells were significantly more resistant to ER stress and apoptosis, clearly showing a reduction in the activation of GRP78/BiP, ERO1α, and cleaved PARP with respect to untreated and mock transduced cells (Fig. 3a). Endoplasmic reticulum stress and UPR induction may also participate in the progress of cellular senescence [47]. Thus, we evaluated the senescence marker p21 [48] in response to ER stress induction (Fig. 3a). Expression of p21 was under the detection limit both in control and PDI-overexpressing cells, regardless of ER stress induction by tunicamycin treatment. Conversely, inhibition of the proteasome by MG132 treatment induced p21 expression, in accordance with the induction of a senescence-like phenotype observed in primary human fibroblasts [49]. Interestingly, MG132-induced upregulation of p21 in PDI-overexpressing cells was reduced compared to untransduced cells, suggesting a possible beneficial effect of PDI expression in reducing the induction of senescence.

Pharmacological preconditioning might represent an additional strategy to confer ER stress resistance to muscle cells. We therefore also analyzed the effect of preconditioning C2C12 cells with quercetin, which has been characterized as an inhibitor of ER stress [36]. We observed by immunoblot analysis that quercetin pretreatment induced a reduction in both GRP78/BiP and cleaved PARP in tunicamycin-treated C2C12 cells, thus reestablishing ER homoeostasis (Fig. 3b). Further supporting our previously reported data, we could observe, in PDI-overexpressing cells, a reduced induction of both GRP78/BiP and cleaved PARP upon ER stress induction, with respect to untransduced cells (Fig. 3b).

Moreover, by cell proliferation assay we confirmed that PDI-overexpressing cells were significantly more resistant to ER-induced cell death with respect to untransduced controls (Fig. 3c). In addition, we determined that quercetin pretreatment partially protected C2C12 cells from tunicamycin-induced cell death, thus confirming that activation of apoptotic cell death can be mitigated by pharmacological modulation of ER stress. Interestingly, PDI overexpression conferred improved cell survival against ER stress also in combination with quercetin treatment (Fig. 3c). Taken together, these data indicate that the modulation of PDI has an antiapoptotic role in muscular cells promoting ER stress resistance.

### Protein disulfide isomerase overexpression promotes engraftment of human myoblasts in *mdx* mice

PDI is expressed at low basal levels in skeletal, cardiac, and smooth muscle cells [50]. To achieve a more robust PDI expression, we transduced human primary myoblasts with adenoviral constructs encoding both GFP and PDI or GFP only, as described previously [30]. At the same time, cells were also transduced with a lentiviral vector expressing the firefly luciferase gene under the control of a constitutive promoter (Lenti-Luc) [37]. When culture medium was switched from maintenance to differentiation medium the transduced cells promptly differentiated into contractile multinucleated skeletal myotubes, indicating that viral-mediated gene transfer of GFP or PDI-GFP and luciferase does not alter the differentiative ability of human primary myoblasts.

**Fig. 4:**
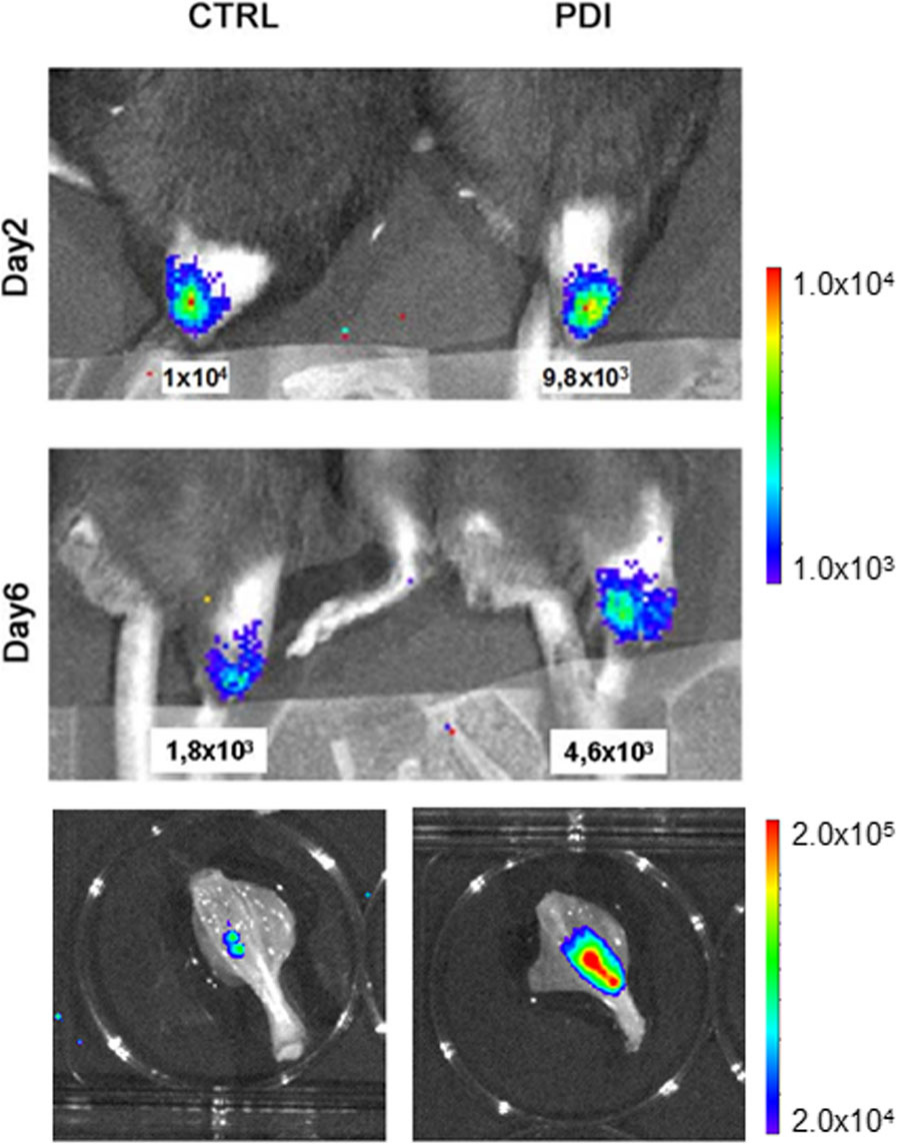
Monitoring of luciferase-positive human primary myoblasts overexpressing PDI transplanted in *mdx* mice. Luciferase-positive human myoblasts (1.0 × 10^5^ cells) transduced either with GFP (CTRL) or PDI-GFP (PDI) were injected into tibialis anterior muscle of 2-month-old, immunosuppressed, *mdx* mice. Engraftment of luciferase-expressing cells monitored from day 1 to day 7 by in vivo bioluminescent imaging and signals in selected area of interest quantified using Living Image software (top panels). At sacrifice, hindlimbs were excised and imaging performed ex vivo (bottom panels). Color bars indicate relative bioluminescent signal intensities from lowest (blue) to highest (red). Values expressed in photons per second per square centimeter per steradian (photons/s/cm^2^/sr). We determined no difference at earliest time point, while signals detected both by in vivo and ex vivo imaging 1 week after implantation were significantly higher in animals receiving PDI-GFP-expressing cells versus mice receiving control cells. Figure shows a representative animal per group (*n* = 5). CTRL control, PDI protein disulfide isomerase

**Fig. 5:**
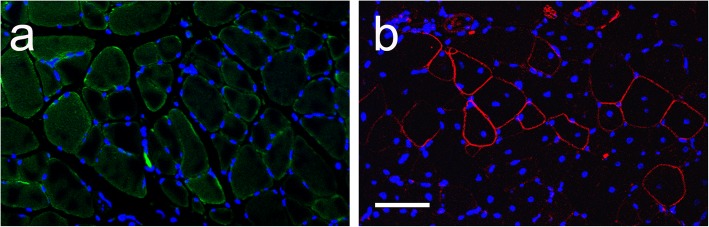
Engraftment of luciferase-positive, PDI-expressing human cells into *mdx* muscle generating dystrophin-expressing fibers. Immunohistochemistry analysis of (**a**) firefly luciferase (green) and (**b**) human dystrophin (red) expression in representative serial sections of tibialis anterior muscle obtained from *mdx* mouse after intramuscular administration of human myoblasts expressing luciferase and PDI. Nuclei visualized by Hoechst staining (blue). Scale bar 50 μm

Human myoblasts (1.0 × 10^5^ cells) were directly injected into the tibialis anterior muscle of 2-month-old *mdx* mice, treated with a daily injection of the immunosuppressant FK-506, beginning on the day of transplant. Engraftment of luciferase-expressing cells was monitored from day 1 to day 7 by in vivo bioluminescent imaging. After 1 week, the persistence of living cells, as measured by bioluminescence, was approximately four times higher (*p* < 0.05) in animals receiving PDI-GFP-expressing cells versus mice receiving control cells expressing GFP only (Fig. 4). At necropsy, tibialis muscles including regions emitting bioluminescence as assessed by ex vivo BLI imaging (Fig. 4) were collected and processed for further analysis. The presence of luciferase-expressing cells in muscle sections from animals administered PDI-expressing, luciferase-positive myoblasts was confirmed by immunohistochemistry analysis using anti-firefly luciferase antibodies (Fig. 5a). Moreover, immunofluorescence detection of dystrophin protein in muscle sections of transplanted animals indicated that PDI-expressing human cells efficiently engrafted into the muscle, generating dystrophin-expressing fibers (Fig. 5b). Thus, overexpression of PDI seems to confer a survival advantage on transplanted primary myoblasts administered into *mdx* mice.

### Transplantation of protein disulfide isomerase-overexpressing cells alleviates hindlimb ischemic damage

**Fig. 6:**
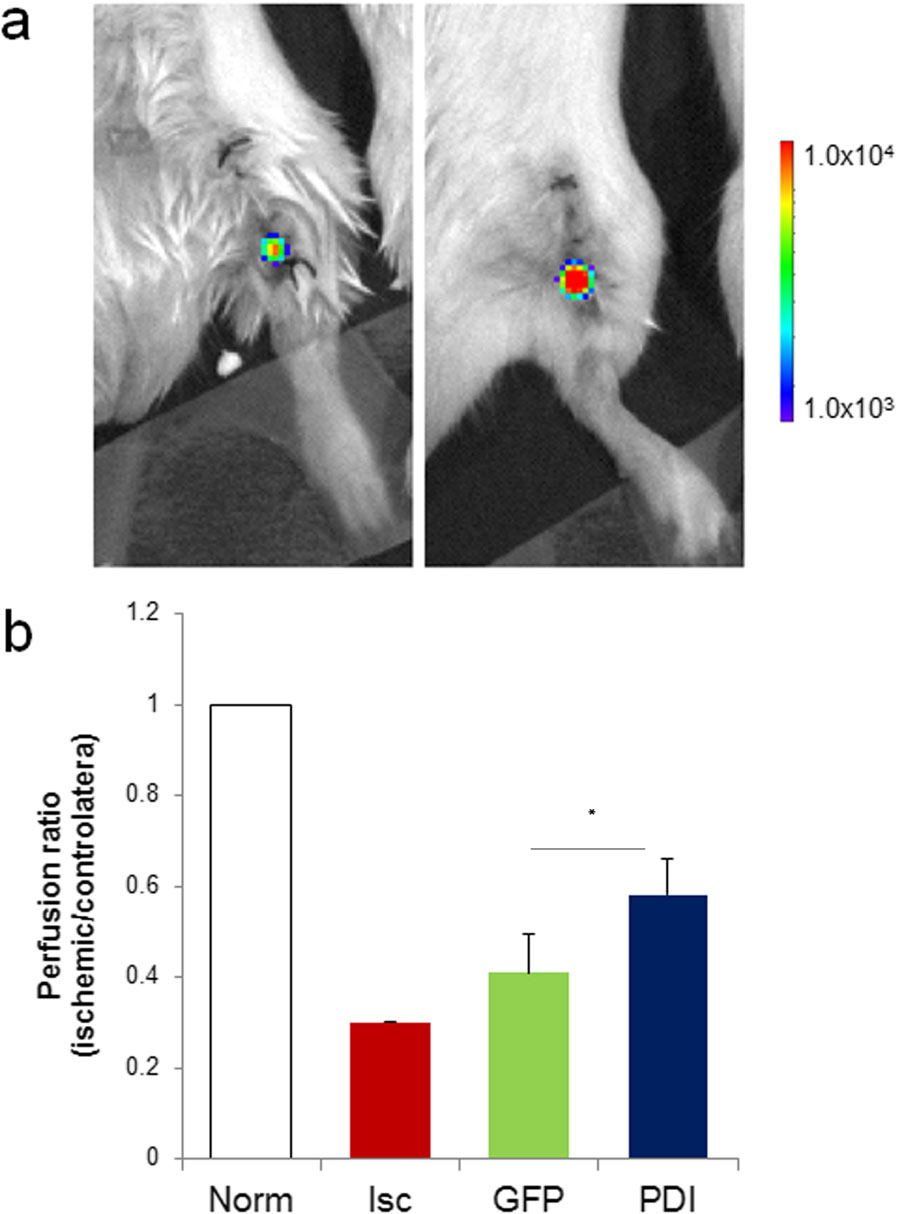
Transplantation of human endothelial cells overexpressing PDI into mouse model of hindlimb ischemia. **a** In vivo bioluminescence analysis of representative mice undergoing experimental ischemia that received transplants of luciferase-expressing human endothelial cells expressing either GFP (CTRL) or PDI and GFP (PDI). Color bar indicates relative bioluminescent signal intensities from lowest (blue) to highest (red). **b** Laser Doppler scanning of blood flow over hindlimbs 1 week after critical limb ischemia. Quantification of measurements expressed as ischemic vs contralateral nonischemic whole limb perfusion ratios in untreated (Isc) and experimental groups administered with GFP (CTRL) and PDI-GFP (PDI)-expressing cells (*n* = 5). Perfusion ratio before ischemia (Norm) shown as reference. *p < 0.05. GFP green fluorescent protein, PDI protein disulfide isomerase

**Fig. 7:**
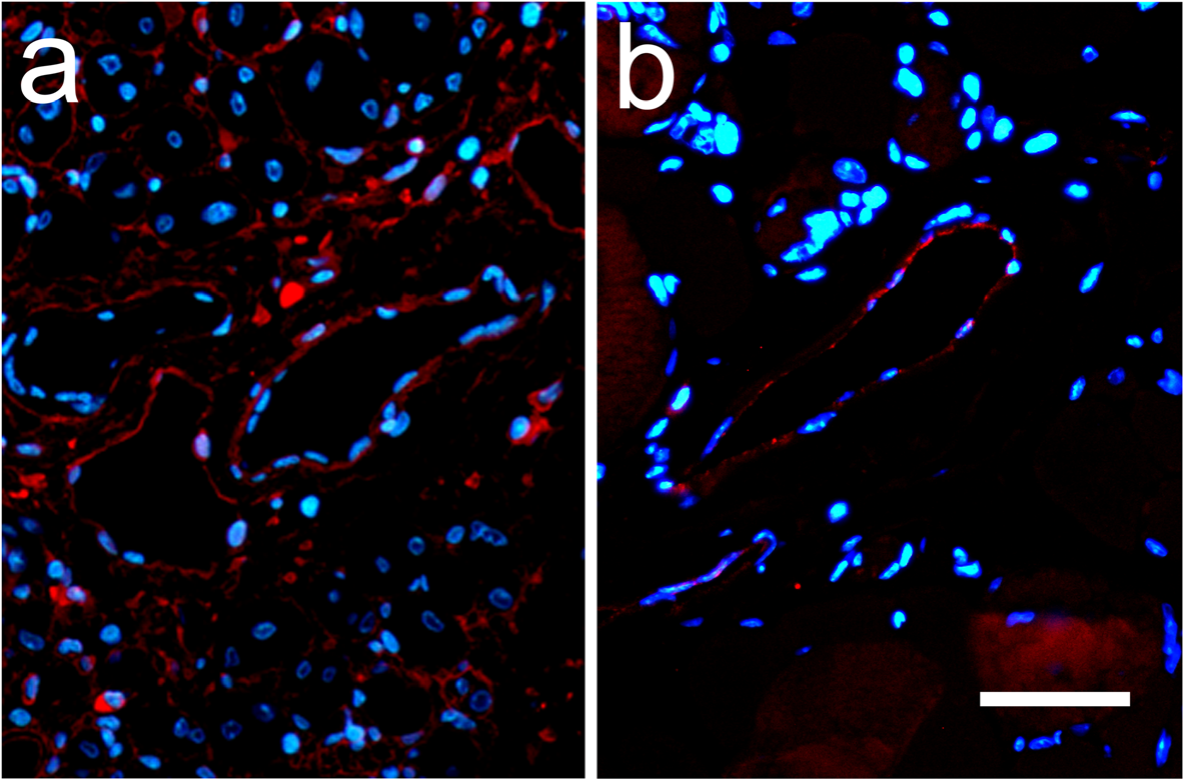
Engraftment of luciferase-positive PDI-expressing human cells into vascular endothelial layer after hindlimb ischemia. Representative fluorescence images obtained with antibodies against (**a**) CD31 (red) and (**b**) PDI (red) in representative sections obtained from mouse receiving human endothelial cells expressing luciferase and PDI-GFP, 1 week after induction of ischemia. Nuclei visualized by Hoechst staining (blue). Scale bar 50 μm

ER stress has been linked to angiogenesis impairment [51] and to endothelial cell dysfunction [52, 53]. Interestingly, PDI is specifically upregulated in endothelial cells to contribute to their ability to tolerate hypoxic stress [54]. On these bases, we assessed whether PDI overexpression can promote therapeutic angiogenesis supporting the survival of transplanted endothelial cells in a mouse model of hindlimb ischemia [55]. Human dermal white adipose tissue represents a convenient source of cells expressing the endothelial-specific marker platelet endothelial cell adhesion molecule (PECAM-1), also known as cluster of differentiation 31 (CD31), able to form tubular-like structures on a Matrigel assay in vitro and to promote angiogenesis in vivo [34, 56]. We engineered primary human adipose tissue-derived CD31^+^ endothelial cells to express firefly luciferase in order to perform cell tracking studies by noninvasive BLI. In addition, cells underwent adenoviral viral-mediated gene transfer of either GFP or PDI-GFP. Hindlimb ischemia was induced by removal of the femoral artery [33], and then 2.5 × 10^5^ cells expressing luciferase and GFP or PDI-GFP were delivered into the tibialis anterior muscle of the experimental animals. Bioluminescent imaging performed 1 week after the transplant showed increased emission in animals receiving PDI-expressing cells. The cell transplantation procedure improved whole limb perfusion measured by laser color Doppler analysis performed 1 week after transplant in comparison with mice not receiving the treatment (Fig. 6b), in accordance with a previously described proangiogenic ability of adipose tissue-derived cells in a hindlimb ischemia model [57]. Remarkably, cell transplantation of cells overexpressing PDI displayed further enhanced vascular regenerative ability, compared to animals receiving control cells (Fig. 6b). Moreover, immunohistochemical analysis performed on tissue samples collected at necropsy confirmed the presence of PDI-GFP-positive cells in the vascular endothelial layer at the site of transplant, identified by specific CD31 expression (Fig. 7). Collectively, these data support the hypothesis that preconditioning vascular cells by PDI gene transfer might support survival of cells administered in ischemic tissue promoting therapeutic angiogenesis.

## Discussion

Cell therapy represents an emerging approach for the treatment of different pathologies including muscular degenerative diseases and cardiovascular pathologies [58]. Cell therapy positive effects are attributable to both cell restoration eliciting functional tissue repair and paracrine action associated with production of growth factors, cytokines, and extracellular vesicles that promote the endogenous mechanisms of tissue regeneration. So far, several cell therapy clinical trials have suffered from limited or transient efficacy, mainly due to poor survival of transplanted cells soon after in vivo delivery [58]. Transplanted cell loss may be initiated by different events including: anoikis, due to the need to detach anchorage-dependent cells from their substrate for injection; inflammation-related factors, such as exposure to cytokines, natural killer cells, and free radicals at the site of transplant; and oxygen and nutrient shortage, due to the absence of a vasculature network within the injected cell clumps [59]. Perturbations of cellular redox regulation and nutrient deprivation can cause accumulation of unfolded proteins generating ER stress [60].

Skeletal and cardiac muscle fibers are characterized by the presence of the sarcoplasmic reticulum, a specialized network of ER that regulates protein homeostasis and calcium concentration [61]. In response to environmental and genetic factors causing ER stress, muscle cells activate the UPR pathways that play pivotal roles in muscle stem cell homeostasis and in muscle regeneration [62]. Low ER stress, eliciting an adaptive UPR response, promotes resistance to a subsequent pathological insult: a process named ER hormesis [63]. UPR response is essential in regulating satellite cell function during skeletal muscle regeneration [64]; remarkably, ER stress-surviving cells differentiate more efficiently into myotubes [65]. On the other hand, chronic ER and UPR response contribute to the pathogenesis of inflammatory myopathies and genetic diseases characterized by progressive muscle degeneration and weakness [61]. Indeed, some ER stress markers, including GRP78, PERK, eIF2a, IRE1, and sXBP1, are upregulated into skeletal muscle from dystrophic mice [45]. In addition, elevated ER stress is exacerbated during aging, due to the progressive reduction of the expression of ER chaperones in different tissues, including the skeletal muscle [66]. Accordingly, chemical chaperone therapy has been tested in dystrophin-deficient *mdx* mice in order to reduce the effects of chronic ER stress [46]. We determined that PDI expression is increased in the tibialis anterior muscle of *mdx* mice, compared to wild-type control (Fig. 1), possibly acting to alleviate ER stress and restore ER homeostasis. We postulated that elevated ER stress in recipient dystrophin-deficient muscle might impair engraftment of transplanted cells in cell-based therapeutic intervention to promote muscular regeneration. Therefore, raising ER stress resistance in transplanted cells might be instrumental in improving cell engraftment in cell therapy procedures. We established that overexpression of PDI promotes survival of transplanted cells to ER stress they face upon transplantation in dystrophin-deficient muscle, enhancing their regenerative potential.

Critical limb ischemia is characterized by markedly reduced blood flow to the extremities and is the most severe and frequent form of peripheral artery disease. Cell therapy represents a promising therapeutic approach for vascular tissue regeneration for ischemic diseases [67]. However, clinical trials indicate consistently modest long-term improvements due, at least in part, to poor survival of transplanted cells [68, 69]. Oxidative stress and ROS generation are essential elements of ER stress [70], induced by physiological stimuli such as hypoxia, glucose, and amino acid deprivation which are critically involved in the early phases of engraftment upon cell transplant [59]. In addition, ER stress has been linked to angiogenesis impairment [51] and endothelial cell dysfunction [52, 53]. Interestingly, PDI is specifically upregulated in endothelial cells to contribute to their ability to tolerate hypoxic stress [54]. Moreover, we have previously determined that PDI protects the heart against ischemic damage [30]. In addition, upregulation of PDI in response to brain ischemia preserves hippocampal cells from apoptosis [71]. Additionally, the use of chemical chaperones mimicking the function of molecular chaperones [72] alleviates ischemia/reperfusion injury [73].

Some cell types, like bone marrow-derived mesenchymal stromal cells, are prone to senescence rather than apoptosis after extensive stress [74], while in others, such as in endothelial cells, ER stress preferentially contributes to apoptosis but not to senescence [75]. PDI expression is decreased in senescent dermal fibroblasts [76] and fetal lung fibroblasts [77], and is lower in stress-induced premature senescent fibroblasts compared to replicative senescent fibroblasts [78]. Conversely, PDI expression is increased in senescent umbilical vein endothelial cells [47, 79, 80]. Moreover, it has been recently described that in endothelial cells PDI has a thiol reductase activity for the dynamin-related protein (Drp1) which regulates mitochondria fission. Therefore, elevated PDI expression should support normal mitochondrial dynamics and endothelial function limiting endothelial cell senescence in the context of pathological conditions such as diabetes mellitus [81].

Collectively, this evidence suggests that PDI plays a protective role in degenerative diseases, in brain ischemia, and in hypoxia, characterized by accumulation of unfolded or misfolded proteins and ER stress. On these bases, we propose that genetic manipulation of transplanted endothelial cells in order to endure ER stress might be instrumental in increasing cell engraftment, promoting the functional recovery by supporting angiogenesis and, consequently, the efficacy of cell therapy for ischemic disease. Here we provide evidence in two relevant models of tissue regeneration, namely muscular degeneration induced by dystrophin deficiency and hindlimb ischemia, that overexpression of PDI in transplanted cells is beneficial for promoting cell survival counteracting ER stress, consequently maximizing the therapeutic benefit of muscular and vascular tissue cell therapy.

## Conclusions

Increasing transplanted cell survival by ex vivo PDI gene transfer may be a novel approach to circumvent the poor persistence after implantation, maximizing the therapeutic benefit of cell therapy for muscular and vascular tissue regeneration.

## Abbreviations

BLI: Bioluminescent imaging
CD31: Cluster of differentiation 31
DMD: Duchenne muscular dystrophy
ER: Endoplasmic reticulum
GFP: Green fluorescent protein
PDI: Protein disulfide isomerase
ROS: Reactive oxygen species
UPR: Unfolded protein response
